# Reducing the uncertainty induced by spatial aggregation in accessibility and spatial interaction applications^☆^

**DOI:** 10.1016/j.jtrangeo.2017.04.001

**Published:** 2017-05

**Authors:** Marcin Stępniak, Chris Jacobs-Crisioni

**Affiliations:** a Institute of Geography and Spatial Organization, Polish Academy of Sciences, ul. Twarda 51/55, 00-818 Warsaw, Poland; b European Commission, Joint Research Centre, Directorate for Growth and Innovation, Territorial Development Unit, Via E. Fermi 2749, 21027 Ispra, Italy

**Keywords:** Accessibility, Distance decay, MAUP, Scale, Spatial interaction

## Abstract

Analyses of spatial interaction are to some degree plagued by uncertainty regarding the impact of spatially dispersed interaction masses within zones on travel times. In this paper, interaction-weighted travel times are computed from a matrix between regularly distributed points at fine resolution, and used together with secondary data to improve estimates of interaction weighted travel time based on commonly applied methods. The paper proposes a method for computing intra-zonal, interaction weighted travel times that is considerably less sensitive to spatial aggregation than existing approaches, and demonstrates that population-weighted centroids are to be preferred over geographically-weighted centroids.

## Highlights

• We focus on methods to reduce scale dependencies in estimation of travel time.
• Harmonic-mean methods are used to obtain benchmark travel times and distances.
• Methods are introduced to substantially improve intra-zonal travel time accuracy.
• Mass-weighted centroids should be used to compute extra-zonal travel time.
• Errors in extra-zonal travel times are largely zone-specific, but no useful penalty variable is found.

## 1 Introduction

Spatial interaction models (SIMs) and measures of interaction opportunity are important workhorses in analyses of transport and trade. Considerable effort is still being put into their methodological advancement (de Vries et al., 2002, de Vries et al., 2001, Patuelli and Arbia, 2013). One problem with these methods is caused by the inherent uncertainties and biases arising from using spatially aggregated interacting masses (Horner and Murray, 2002, Kwan and Weber, 2008). The uncertainties and biases caused by spatially aggregated data imply that findings obtained from one set of areal units are not necessarily valid for areal units with different shapes or another spatial resolution. This is commonly referred to as the Modifiable Areal Unit Problem (MAUP), and it is known as a particularly persistent issue that, at best, can only be partially mitigated ((Arbia, 1989, Jacobs-Crisioni et al., 2014, Niedzielski et al., 2013, Openshaw, 1984). Spatially aggregated data are used in spatial interaction studies primarily because of data availability and computational constraints. Data constraints exist because the analysis has to ‘fit’ into statistical areas (Goodchild, 1979). Computational constraints exist because of the two-dimensional nature of analyses of spatial interaction, which implies that increasing the number of units leads to exponentially larger origin-destination matrices. Thus, although very fine resolution population raster datasets are increasingly available, large units such as LAU-2 or NUTS-3 zones are often used to model accessibility (e.g. Moya-Gómez and García-Palomares, 2015, Ortega et al., 2014, Spiekermann et al., 2013).

At the root of the MAUP is the issue that the underlying distribution of the process being modelled cannot be precisely known when the areal units that are analysed do not represent the individual entities that are being studied. Gotway and Young (2002) therefore refer to the MAUP as a change of support problem. The MAUP presents itself through scale and shape effects, which affect statistical results through the spatial resolution and the spatial form of areal units respectively. To mitigate the MAUP, some advocate the use of methods that do not depend on spatial aggregation (Kwan, 1998, Kwan and Weber, 2008, Tobler, 1989), but these methods typically require substantial additional data, which are often unavailable. Thus, instead of analysing interactions between individual masses, the geographical centre (hereafter: ‘geographic centroid’) of a zone is typically presumed to represent the spatial distribution of all individual masses in that zone (Hodgson et al., 1997), and then used to compute degrees of separation from other centroids. Hillsman and Rhoda (1978) identified two types of aggregation error related to that approach: one related to the misestimation of degrees of separation from destinations in other areal units (i.e. extra-zonal travel, described as Source A errors); and one related to the misestimation of degrees of separation in the special case that the origins and destinations are represented by the same point in space (i.e. intra-zonal travel, described as Source B errors).^1^ So far, attempts to resolve the MAUP in SIMs have been unsuccessful. Solutions have been proposed in the location-allocation literature (Current and Schilling, 1987, Hodgson et al., 1997), but those are not applicable to spatial interaction applications because of the presence of multiple destinations and distance decay in those applications. Batty and Sikdar, 1982a, Batty and Sikdar, 1982b attempted to rectify the MAUP through understanding its implications on entropy. They essentially concluded that the MAUP is a persistent issue in one-dimensional approaches; let alone in two-dimensional approaches such as SIMs. More recently, the growing availability of fine-resolution data has allowed studies of the impact of the MAUP in one-dimensional empirical applications, with the general conclusion that statistical results presumably depend more on model specification than on the MAUP (Amrhein, 1995, Briant et al., 2010). Recently, Jacobs-Crisioni et al. (2014) concluded that MAUP effects consist of a structural and a chance element, and that at least the structural elements in MAUP effects can be reduced.

The objective of this paper is to explore and reduce the impacts that spatial resolution has on the results of accessibility and spatial interaction models, without substantially increasing data requirements, computational complexity or analyst workload. Methods are sought to use secondary data sources in order to most accurately represent, at the aggregate level, the degrees of separation overcome by interactions between individual masses. This is done using travel times obtained from modelled network impedances, as is frequently done in the relevant literature. We find that even in completely controlled accessibility and SIM applications (i.e. models in which interaction behaviour is assumed to be perfectly homogeneous), structural aggregation effects exist because of inaccuracies in the estimation of degrees of separation within and between areal units. In the remainder of this paper, degrees of separation are discussed as distances or travel times, but our findings no doubt hold for other measures of separation as well – for instance, generalised travel costs.

**Fig. 1:**
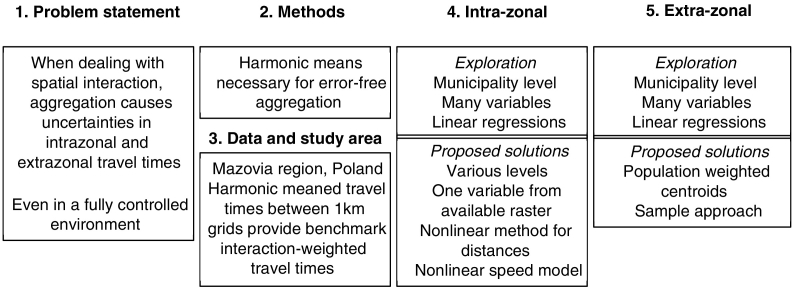
Structure of this paper.

The structure of this paper is outlined in Fig. 1. In Section 2 we investigate travel time between two areal units, which we consider a descriptive characteristic of geography; and interaction-weighted travel time between two areal units, which we consider a characteristic of the interaction between the spatially dispersed masses that are represented by these areal units. Besides measuring a degree of (spatial) separation, the latter includes a propensity to interact between the masses at the origin and destination. Section 2 proceeds to demonstrate that inaccurate assumptions on degrees of separation between areal units are the cause of structural aggregation errors that emerge even in fully controlled spatial interaction analyses. We subsequently use harmonic mean travel times from a very fine resolution origin-destination matrix to obtain error-free interaction-weighted travel times between various aggregation schemes. Those are then used as benchmark values to search methods to obtain the most accurate, scale-insensitive interaction-weighted travel times between areal units given the restrictions stated in the objective. The study area, aggregation schemes and data used are described in Section 3. The harmonically meaned travel times were used as benchmark values for obtaining more accurate intra-zonal travel times (i.e., reduce source B errors). This is shown in Section 4. Furthermore, the available benchmark values were used to explore methods for reducing extra-zonal (source A) errors. This is shown in Section 5. Both analytical sections (i.e. 4, 5) start with the state-of-the-art by summarising existing approaches to intra-zonal and inter-zonal travel time estimations, respectively. Section 6 offers general conclusions and thoughts on follow-up research. Lastly, it is worth mentioning that all the data used is freely available (Jacobs-Crisioni and Stepniak, 2017).

## 2 Aggregating travel times in spatial interaction applications

As noted in the last section, we need to separate point-to-point travel time, and interaction-weighted travel time. The first one is expressed by a simple value *t*_*ij*_, which describes the time-distance that separates a pair of origin-destination points. In this case the mass or attractiveness of a destination does not have any impact on the aggregation procedure and need not be considered. This study focuses on cases in which travel times are expressed as a characteristic of spatial interaction, such as in SIMs. These SIMs typically include at least a measure of propensity to produce interactions at a place of origin, a measure of propensity to attract these interactions at available destinations, and a measure of the degree of separation that any travel between origin and destination needs to overcome (Alonso, 1978, Wilson, 1967). An unconstrained SIM takes this form:

(1) $$F_{\mathrm{ij}}=\frac{P_{i}P_{j}}{f\left(t_{\mathrm{ij}}\right)},$$

in which the flows *F* between origins *i* and destinations *j* depend on population size *P* and travel time *t*. An impedance function *f* is then typically used to impose an interpretation of travel time valuation in the decisions in the model. Potential accessibility measures are derived from SIMs and widely used in accessibility and economic analyses (for an overview see Geurs and van Wee, 2004 and Östh et al., 2014). That measure reflects the potential number of interactions from or to one place. An unconstrained potential accessibility measure takes this form:

(2) $$A_{i}=\sum_{j=1}^{n}\frac{P_{j}}{f\left(t_{\mathrm{ij}}\right)}.$$

Constrained versions of SIMs and potential accessibility are also available in the literature (see e.g.: Alonso, 1978, Geurs and Ritsema van Eck, 2003). For the sake of simplicity these are not treated in this paper.

Any act of spatial aggregation consists of summing or averaging values from a fine to a coarser spatial resolution (Arbia, 1989). Invoke subset of origins *a* located within unit *I* (*a* ∈ *I*), and subsets of destinations *b* in the unit *J* (*b* ∈ *J)*. Consider *a* and *b* populated by *P*_*a*_ and *P*_*b*_ respectively, and consider *t*_*ab*_ as travel times between the origins and destinations. Under aggregation, summing the people by e.g. $P_{j}=\sum_{b\in J}^{n}P_{b}$ is straightforward. However, we will also need to average *f*() and ensure that it represents the aggregate outcomes of that function for individual interactions between *i* and *j*. This, unfortunately, is less straightforward. Let's produce an easy example with four people living in different destinations that are respectively 4, 6, 7 and 7 min away from the origin considered. That variation in travel time may be due to the geographical locations of the destinations or because different paths are used to reach those destinations, but the source of travel time variation is not relevant here. Distance decay is linear so that $f\left(t_{\mathrm{ab}}\right)={t_{\mathrm{ab}}}^{1}$; given that *f*(*t*_*ab*_) always yields *t*_*ab*_ in this example, we can ignore the implications of nonlinear distance decay in this introductory example and come back to it later. As a point of interest *a,* the population-weighted arithmetically averaged travel-time (computed as $\sum_{b\in J}^{n}w_{b}x_{b}/\sum_{b\in J}^{n}w_{b}$ with the number of people indicated by weight *w* and travel time indicated by *x*) would be 6 min. Thus, if we compute results for a potential accessibility measure *A* in the disaggregated and aggregated case:

(3) $$A_{a}=\left(\frac{1}{7}+\frac{1}{7}+\frac{1}{6}+\frac{1}{4}\right)\neq \sum \left(P_{b}/\frac{\sum P_{b}t_{\mathrm{ab}}}{\sum P_{b}}\right)=\frac{4}{6}.$$

Here, the arithmetically averaged travel time on the right does not accurately represent average interaction travel times, and the real value of potential interactions A, $\frac{59}{84}$, cannot be computed by summing people and arithmetically averaging the travel times to those people. In fact the use of arithmetical averages here would consistently yield an overestimation of travel times in the interactions measured, as is, for example, reflected in the findings obtained by Stępniak and Rosik (2015). The problem here is that the computed unit sums potential interactions, which are rates. As is always the case when averaging rates, harmonic means (computed as weighted harmonic means $\sum_{b\in J}^{n}w_{b}/\sum_{b\in J}^{n}\frac{w_{b}}{x_{b}}$) are required. In this example, the weighted harmonic mean travel times of our example would yield roughly 5.695. Thus:

(4) $$A_{a}=\left(\frac{1}{7}+\frac{1}{7}+\frac{1}{6}+\frac{1}{4}\right)=\sum P_{b}/\frac{\sum P_{b}}{\sum \frac{P_{b}\;}{t_{\mathrm{ab}}}}=\left(4/\frac{4}{\left(\frac{1}{7}+\frac{1}{7}+\frac{1}{6}+\frac{1}{4}\right)}\right)≅\frac{4}{5.695}.$$

**Fig. 2:**
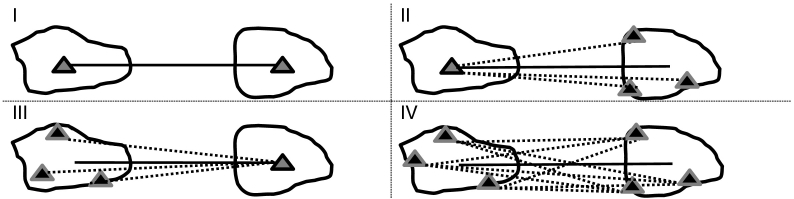
Schematic representation of travel time definitions between two zones. Dark outlined triangles signify centroids; light outlined triangles signify spatially distributed people. Solid lines signify the travel times obtained, while dashed lines indicate the real situation represented by the travel times obtained.

It can be easily verified that the result of this function is identical to $\frac{59}{84}$. In this example, the difference between the harmonic and arithmetic means is caused by the fact that, due to distance decay in interactions, the person living closest contributes more than proportionally to total interaction opportunity. This brings us back to the distinction between descriptive travel times and interaction-weighted travel times. The first category (category I in Fig. 2) essentially describes the difficulty of overcoming geography between pairs of points located within two separate areal units, without interaction, or at least without the spatial dispersion of interacting masses within the zone. In this case, a node represents the whole spatial unit, concentrating all its ‘mass’ (e.g. population, jobs etc.) within the point. In the case of spatial interaction with interacting masses that are dispersed within a zone, harmonic means become relevant. We identify three different travel time cases. Categories II and III describe cases where potential interaction between one point and spatially dispersed interacting masses only occurs at the origin or destination; these categories are relevant for accessibility measures that aim to measure the *territorial* distribution of interaction opportunity, such as studied by Vickerman et al. (1999) or applied in the LUISA model (Jacobs-Crisioni et al., 2016). Category IV describes the potential interaction between spatially dispersed interacting masses in both the origin and destination, such as is commonly the case in SIMs or accessibility measures that aim to measure the *social* distribution of opportunity for interaction.

In category I, the intra-zonal distribution of activities is not relevant, as there is only one travel time between one pair of points. However, in the case of interaction, travel times are subject to the interaction between at least *P*_*a*_ or *P*_*b*_ and *t*_*ab*_. As is shown above (Eq. (4)), this requires the use of harmonic means during aggregation:

(5.1) $$t_{\mathrm{ij}}^{\mathrm{II}}=\frac{\sum_{b\in J}^{n}P_{b}}{\sum_{\mathrm{ba}\in \mathrm{JI}}^{n}\frac{P_{b}}{t_{\mathrm{ab}}}};$$

(5.2) $$t_{\mathrm{ij}}^{\mathrm{III}}=\frac{\sum_{a\in I}^{n}P_{a}}{\sum_{\mathrm{ab}\in \mathrm{IJ}}^{n}\frac{P_{a}}{t_{\mathrm{ab}}}};$$

(5.3) $$t_{\mathrm{ij}}^{\mathrm{IV}}=\frac{\sum_{b\in J}^{n}P_{a}P_{b}}{\sum_{b\in J}^{n}\frac{P_{a}P_{b}}{t_{\mathrm{ab}}}}.$$

It is important to note here that the above aggregation procedures also yield values for the special case that *a* and *b* are both located in the same larger areal unit, so that this approach also yields reliable interaction-weighted distances and travel times within zones. This will be exploited in Section 4 to investigate intra-zonal travel time models.

As noted before, the previously presented equations only hold in the case of linear distance decay. It can easily be verified that the values of interaction-weighted travel times depend on what impedance function is imposed. Power and exponential impedance functions are commonly applied in spatial interaction applications (Kwan, 1998, Reggiani et al., 2011). We therefore compute the benchmark values in the cases of power (Eq. (6.1)) and exponential (Eq. (6.2)) distance decay, using the following inverse functions:

(6.1) $$t{\left(\gamma \right)}_{\mathrm{ij}}^{\mathrm{IV}}={\left(\sum_{b\in J}^{n}P_{a}P_{b}/\sum_{b\in J}^{n}\frac{P_{a}P_{b}}{{t_{\mathrm{ab}}}^{\gamma }}\right)}^{1/\gamma }$$

(6.2) $$t{\left(\delta \right)}_{\mathrm{ij}}^{\mathrm{IV}}=\ln\left(\sum_{b\in J}^{n}P_{a}P_{b}/\sum_{b\in J}^{n}\frac{P_{a}P_{b}}{\exp\delta \;t_{\mathrm{ab}}}\right)/\delta$$

A derivation of the above equations is given in Appendix A. Different values of *γ* and *δ* were computed in order to provide useful functions in a wide range of conditions. These values have been obtained from the literature, supplemented with strategic selections by the authors, and are listed in Section 4. They are used to investigate models of intra-zonal travel distances in that section. Section 5 focuses entirely on travel times *t*(*γ*)_*ij*_^*IV*^ with *γ* = 1.

Interaction-weighted travel times, aggregated using harmonic means, will be used as benchmark values. From here on, we use both terms, i.e. interaction weighted-travel times and benchmark values, interchangeably. For the sake of simplicity, in the main text the paper only discusses the results from comparisons with category IV benchmark values. The results from comparisons with category II benchmark values are equal to the results of category III, but differ quantitatively from the category IV results. Where necessary, the results relevant to category II and III are given in Appendix B.

## 3 Study area, aggregation schemes and data

### 3.1 Study area and travel time data

The subject of this study is Mazovia, a relatively large and diverse region in the central part of Poland, with the national capital Warsaw at its centre. To compute benchmark values, fine resolution population levels and travel times are used, taken from Stępniak and Rosik (2015). In that data, origins *a* and destinations *b* consist of 27,632 inhabited points on a 1 km regular lattice, adapted from GEOSTAT 2006. Furthermore the data used contain travel times *t*_*ab*_ in minutes and Euclidean distances *d*_*ab*_ in kilometres. Travel times were obtained using shortest path methods on comprehensive road network data (70,000 edges approximately).

### 3.2 Aggregation schemes

Various sets of areal units, all based on administrative boundaries, provide the origin and destination units *i* and *j* that are necessary to explore spatial aggregation impacts. Initially the paper repeatedly explores solutions to improve travel time estimation methods using benchmark values between municipalities (LAU-2, *n* = 300), and then proposes solutions that reduce scale dependencies in travel time estimation methods. In order to address the MAUP issue, the proposed solutions are tested on a broader set of areal units that additionally includes ‘*powiats’* (LAU-1, *n* = 38), and even larger units, ‘greater regions’ (*n* = 4). These largest units consist of *powiats* that are combined by the authors into four simply identifiable parts.

**Fig. 3:**
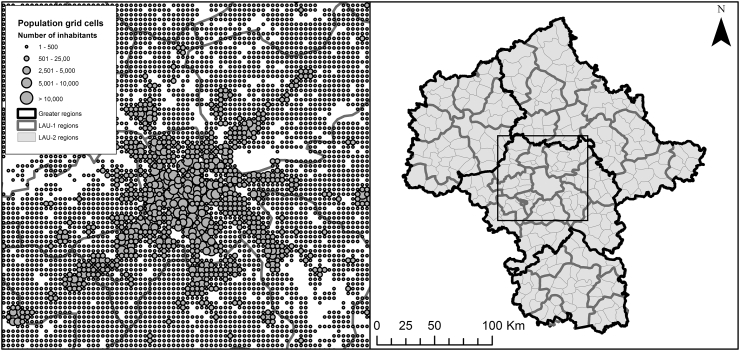
Maps depicting populated grid cells and LAU-1 borders around Warsaw, Poland (left) and the three sets of areal units used in the analysis (right). The frame in the right map indicates the spatial extent of the left map.

Interaction weighted travel times *t*_*ij*_^*IV*^ and Euclidean distances *d*_*ij*_^*IV*^ have been aggregated to all areal unit sets. The potential interaction within a regular lattice point is discarded in this study because the travel times within those zones cannot be known – at least not without taking into account population distribution data at an even finer, computationally impractical resolution. There are, on average, 92 regular lattice points in one LAU-2 unit so that, on average, 92/92^2^ ≅ 0.01 of all observations relevant for intra-zonal distances are ignored. We therefore expect that the omission of this small share of observations does not considerably bias the model results. Table 1 shows descriptive statistics from the units used, including benchmark (i.e. interaction-weighted) travel times and Euclidean distances, given linear distance decay. All areal units used in the study are shown in Fig. 3.

**Table 1:** Areal units used in the study and average benchmark values.

Unit name	N	Interaction-weighted travel time (minutes)		Interaction-weighted Euclidean distance (km)	
		Intra-zonal	Extra-zonal	Intra-zonal	Extra-zonal
Regular lattice points	27,632	–	114.70	–	101.69
LAU-2 (*municipalities*)	300	8.34	110.73	3.38	99.15
LAU-1 (*powiats*)	38	14.68	107.85	6.37	98.15
Greater regions	4	35.59	98.38	17.62	88.29

### 3.3 Secondary variables

Secondary variables were used to search proxy variables that help reduce structural aggregation errors in travel times. The expectation was that aggregation errors come into play because of spatial activity distributions or road connectivity, so the only variables that were selected were those that described these aspects of the zones that were analysed. Only freely available or easily computable variables were selected. We made our selection based on variables commonly used in the literature for internal travel times (cf. Table 3 in the next section), and other variables, which according to our intuition and experience might influence travel time (or distance). These variables (see Table 2) describe spatial characteristics (e.g. area or radius); population levels (population, population density); road network (e.g. network density); a landscape characteristic named average Weighted Urban Proliferation (WUP) (Jaeger and Schwick, 2014); and degrees of urbanisation (Dijkstra and Poelman, 2014). Moreover, other variables are included to observe internal population distributions. Those variables indicate the share of inhabited grid cells in all grid cells and the population's Gini index and Moran's I. Finally, we add the population weighted average Euclidean distance to the zone's gravity-based population centre.^2^ Lastly, using benchmark travel times and Euclidean distances, effective intra-zonal effective travel speeds were computed as *V*_*ij*_ = *d*_*ij*_^*IV*^/(*t*_*ij*_^*IV*^/60). These are expressed in km/h.

**Table 2:** Descriptive statistics of the variables investigated aggregated to the LAU-2 areal unit set.

Variables	Min.	Median	Mean	Max.	SD
Area	5.8	115.0	118.7	517.1	55.9
½ radius	0.68	3.02	2.99	6.41	0.72
Population size	1715	6444	17,480	1,720,000	100,118
Population per square km	18.91	53.91	196.73	4214.66	518.82
Degree of urbanisation	1.00	3.00	2.74	3.00	0.54
Average WUP	0.00	0.03	0.08	1.07	0.14
Intra-zonal effective travel speed	10.60	25.29	24.26	30.56	3.90
Network/km^2^	0.24	0.60	0.64	2.00	0.22
Population Gini index	0.17	0.67	0.65	0.91	0.11
Population Moran's I	− 0.12	0.28	0.30	0.79	0.19
Average distance to population centroids	0.8	3.7	3.5	6.6	1.0

Table note: Observations from LAU-2 units so that *N* = 300.

Fine resolution population data are necessary for some of the variables selected. Such data are, at the moment of writing, becoming available globally with a reasonable level of accuracy (Freire et al., 2016). If necessary, future population distributions can be obtained using methods such as the LUISA model (Jacobs-Crisioni et al., 2016). The availability of data describing other potentially relevant activities might be more limited. Reasonably accurate maps of other activity types can also be obtained using methods such as those in Batista e Silva et al. (Batista e Silva et al., 2013).

## 4 Estimating intra-zonal interaction-weighted travel times

This section is dedicated to reducing so-called source B errors (Hillsman and Rhoda, 1978), which is known as the self-potential problem (Frost and Spence, 1995) in the accessibility literature. This issue arises when the origin and destination are represented by the same point, so that methods to obtain travel time or distance yield zero even when masses are spatially distributed. To overcome this, it is necessary to guess intra-zonal interaction weighted travel time.

### 4.1 Existing methods

Previous studies have shown that the estimation of intra-zonal travel times is not trivial, and self-potential may contribute up to 60% of total accessibility (Condeço Melhorado et al., 2016, Frost and Spence, 1995, Gutiérrez, 2001). The presumably most common solution to this problem is Rich's method to obtain intra-zonal distances from the area of the unit (Frost and Spence, 1995, Rich, 1978), complemented by a guess of intra-zonal travel speeds to obtain travel times. Others derived intra-zonal travel times from the area and shape of a zone (Kotavaara et al., 2011); linked population sizes with travel times (Condeço-Melhorado et al., 2013, Gutiérrez, 2001); or aggregated average travel times from smaller units (Ottaviano and Pinelli, 2006, Salas-Olmedo et al., 2014). An overview of approaches for obtaining intra-zonal travel times is given in Table 3.

**Table 3:** Selected approaches to estimate internal trip lengths (travel time and/or distance).

	Reference	Internal travel distance/time	Speed
Area based (Rich's formula and its modifications)	Frost and Spence (1995)	Various radii (0.33–1.0)	30.4–90.2 km/h
	Baradaran and Ramjerdi (2001)	½ radius	40 km/h
	Gutiérrez et al. (2011)	½ radius	~ Pop. density 20–80 km/h
	Jacobs-Crisioni and Koomen (2014)	½ radius	~ Distance *V* = 10.66 + 13.04ln(*d*_*j*_)
	Stepniak and Rosik (2013)	½ radius	20 km/h
	van Wee et al. (2001)	${\mathrm{Dist}}_{i}=\frac{2^{*}\sqrt{{\mathrm{area}}_{i}}}{3}$	National average 33 km/h
	Kotavaara et al. (2011)	Area & shape of unit	40 km/h
Internal travels	Salas-Olmedo et al. (2014)	Weighted average distance between all units inside the region	–
	Ottaviano and Pinelli (2006)	Weighted average distance between all units inside the region	–
Population based	Condeço-Melhorado et al. (2013)	T: Population: *t*_*ii*_ = 3.4336Ln(*P*_*i*_)-0.8476 12–28 min (sample of Spanish cities)	–
	Gutiérrez (2001)	T: Population: *t*_*ii*_ = 15 ^⁎^ log(*P*^⁎^10)	–

### 4.2 Exploration

The benchmark travel time and Euclidean distance values obtained in Section 3 were instrumental here in verifying the approaches applied in the literature and searching for the most accurate approach for computing intra-zonal interaction weighted travel times. This search focused on two-step methods such as Rich's (1978), in which, first, intra-zonal distances are estimated based on the spatial structure of a zone, and then, to obtain interaction weighted travel times, effective speeds are estimated. With the proposed two-step approach, we can separate a general effect of spatial structure on distances from case-specific effects of the efficiency and quality of transport infrastructure on effective speeds. We expect that the proposed method is the approach that is generally the most useful for analysts dealing with the same challenge in different contexts.

The search for the most accurate method of obtaining intra-zonal travel distances consisted of three stages. In the first stage, all available zone-specific variables were used separately to explain the differences in benchmark intra-zonal distances between LAU-2 units with *γ* = 1. The following linear equation was repeatedly fitted in an OLS approach:

(7) $$d{\left(\gamma =1\right)}_{\left(i=j\right)}^{\mathrm{IV}}=\beta 0+\beta 1X_{i}+\varepsilon_{i},$$

so that the effects of several variables *X* on intra-zonal travel distances are tested separately. The results are shown in Table 4.

**Table 4:** Results of linear models with all the variables investigated, with the most accurate model at the top.

Variable	*β*0 (z-score)	*β*1(z-score)	R^2^	RMSE
Average distance to population centroids	0.571⁎⁎ (12.92)	0.798⁎⁎ (66.42)	0.94	0.214
Frost and Spence ½ radius	1.111⁎⁎ (6.97)	0.762⁎⁎ (14.69)	0.42	0.647
Average WUP	3.685⁎⁎ (82.22)	− 3.646⁎⁎ (− 13.31)	0.37	0.673
Degree of urbanisation	0.889⁎⁎ (4.34)	0.913⁎⁎ (12.45)	0.34	0.689
Area	2.351⁎⁎ (25.05)	0.009⁎⁎ (12.20)	0.33	0.693
Network density	4.539⁎⁎ (33.54)	− 1.796⁎⁎ (− 8.98)	0.21	0.753
Ln (population)	7.431⁎⁎ (15.51)	− 0.450⁎⁎ (− 8.48)	0.19	0.762
Population density	3.527⁎⁎ (74.75)	− 0.001⁎⁎ (− 8.36)	0.19	0.764
Moran's I	3.960⁎⁎ (46.99)	− 1.880⁎⁎ (− 8.00)	0.18	0.770
Gini coefficient	4.029⁎⁎ (13.98)	− 0.981⁎ (− 2.26)	0.02	0.842
Population	3.373⁎⁎ (68.10)	0.000 (1.68)	0.01	0.845

Table note: intra-zonal observations from the LAU-2 matrix so that *N* = 300.

⁎ *p* < 0.05.

⁎⁎ *p* < 0.01.

### 4.3 Proposed solutions

The average distance to the zone's population-weighted centroid (${\overset{-}{\mathrm{dw}}}_{i}$, see footnote 2) proved to be substantially more accurate than the second-most accurate estimator (½ radius). Using the best fitting variable, we extended our analysis in order to obtain results that are independent of scale by fitting nonlinear functions on the data from all 342 of the available areal units combined. Nonlinear functions were used because with OLS, the explanatory variable correlated with the regression residuals indicating that the explanatory variable has a nonlinear impact here. A search of model specifications yielded the following simplest form in which the explanatory variable does not correlate with the regression residual:

(8) $$d_{i=j}^{\mathrm{IV}}=\beta 0^{*}{\bar{\mathrm{dw}}}_{i}^{\beta 1}+\varepsilon_{i}.$$

Eq. (8) has been fitted on the benchmark distances from all the selected impedance functions. The results of that fitting exercise are given in Table 5. The RMSE produced by the model over the entire set of areal units then varies between 0.07 and 0.45 km. The accuracy gain compared to Rich's approach will be discussed later in this section. The results for category II and III travel times differ substantially from the category IV results and are given in Appendix B. This difference is to be expected, given that the benchmark travel time values for those categories depend only on population distributions in one zone, while the values in category IV depend on interaction between the spatially dispersed populations in both the origin and destination.

**Table 5:** Model parameters and evaluation of estimations of internal travel distance.

Impedance function (value)	*β*0 (z-score)	*β*1 (z-score)	R^2^	RMSE (km)	MAPE (%)
Power function (− 1.00)	1.26409⁎⁎ (72.32)	0.76822⁎⁎ (124.32)	0.9896	0.4518	0.0672
Power function (− 1.25)	1.32190⁎⁎ (67.81)	0.68937⁎⁎ (98.50)	0.9882	0.4311	0.0724
Power function (− 1.50)	1.38086⁎⁎ (64.88)	0.61046⁎⁎ (78.75)	0.9874	0.4026	0.0796
Power function (− 1.75)	1.42552⁎⁎ (62.92)	0.53980⁎⁎ (64.29)	0.9869	0.3729	0.0863
Power function (− 2.00)	1.45095⁎⁎ (61.90)	0.48032⁎⁎ (54.12)	0.9869	0.3436	0.0902
Power function (− 2.50)	1.45593⁎⁎ (62.46)	0.39123⁎⁎ (42.14)	0.9877	0.2886	0.0916
Power function (− 4.00)	1.35367⁎⁎ (76.03)	0.25323⁎⁎ (30.84)	0.9922	0.1725	0.0752
Power function (− 8.00)	1.17934⁎⁎ (135.37)	0.13392⁎⁎ (27.49)	0.9976	0.0699	0.0412
Exponential (− 0.006)^b^	1.54929⁎⁎ (160.29)	0.95014⁎⁎ (391.82)	0.9981	0.3567	0.0366
Exponential (− 0.023)^b^	1.65885⁎⁎ (154.38)	0.89276⁎⁎ (339.56)	0.9978	0.3547	0.0372
Exponential (− 0.039)^c^	1.74476⁎⁎ (142.35)	0.84714⁎⁎ (286.87)	0.9974	0.3700	0.0403
Exponential (− 0.068)^d^	1.86810⁎⁎ (125.71)	0.77866⁎⁎ (220.82)	0.9966	0.3921	0.0460
Exponential (− 0.15)	2.06878⁎⁎ (102.81)	0.64735⁎⁎ (135.92)	0.9949	0.4095	0.0564
Exponential (− 0.25)	2.16230⁎⁎ (90.54)	0.54984⁎⁎ (94.88)	0.9936	0.4009	0.0752
Exponential (− 0.289)^a^	2.17544⁎⁎ (87.39)	0.52186⁎⁎ (85.29)	0.9932	0.3956	0.0707
Exponential (− 0.90)	1.97065⁎⁎ (72.05)	0.32677⁎⁎ (39.17)	0.9910	0.3011	0.0881

Table note: intra-zonal observations from all areal units so that *N* = 342. ^⁎^p < 0.05. Exponential function selected by the authors, except values obtained from:

a Haynes et al. (2003).

b Rosik et al. (2015).

c Geurs and Ritsema van Eck (2001).

d Martínez and Viegas (2013).

⁎⁎ *p* < 0.01.

**Fig. 4:**
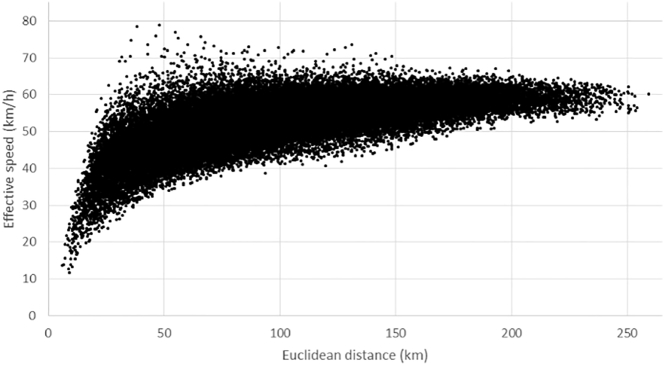
Effective speeds and Euclidean distances according to the LAU-2 based benchmark travel times and distances in the study region.

The above model yields intra-zonal interaction-weighted travel distances and thus describes a zone's spatial structure. To be used in accessibility and SIM applications, an additional step is necessary to obtain, for example, travel times or generalised travel costs. In search of an optimal solution, this paper focuses on a method for obtaining free-flow effective travel speeds, but we emphasise that a more refined approach, which takes for example congestion or travel costs into account, is equally possible. As can be seen in Table 3, a number of approaches to applying travel speeds are available in the literature, however the resulting travel speeds vary substantially depending on the approach chosen. Some studies use travel surveys (Frost and Spence, 1995) or speed profile data (Condeço Melhorado et al., 2016). Others assume that travel speeds are constant for all the zones in the model (Kotavaara et al., 2011), that speeds depend on population characteristics (Gutiérrez, 2001), or that speeds depend on internal travel distance (Jacobs-Crisioni and Koomen, 2014). Our model assumes that effective speeds depend on distance travelled. That model is estimated on the LAU-2 travel time matrix that in any case needs to be computed for the analyses this study intends to support. We find an acceptable fit with an OLS estimation of the following equation:

(9) $$\ln\left(V_{\mathrm{ij}}\right)=\beta 0+\beta 1d_{\mathrm{ij}}^{\mathrm{IV}}+\beta 2\ln\left(d_{\mathrm{ij}}^{\mathrm{IV}}\right)+\beta 3IZ_{i}+\varepsilon_{\mathrm{ij}},$$

in which *d* is well-known and *IZ* is a dummy variable that takes the value of one if *i = j* and zero otherwise. The value distribution on which this function is fitted is shown in Fig. 4. We interpret the relation found between Euclidean distance and effective speed as an abstraction of the fact that detour factors tend to decrease with distance while, simultaneously, the usage of fast infrastructure tends to increase (Table 6). The results of the intra-zonal estimator *IZ* show that intra-zonal observations hardly affect the fitted model, so that this function can be fitted using only extra-zonal travel times.

**Table 6:** Results of fitting of the average travel speed model.

	Coef.	z-score
Constant	2.836⁎⁎	(594.1)
Distance	− 0.001⁎⁎	(− 58.00)
Ln (distance)	0.272⁎⁎	(188.35)
Intra-zonal *IZ*	0.018⁎⁎	(2.80)
N	90,000	
R2	0.63	
RMSE	0.10	

Table note: ^⁎^*p* < 0.05.

⁎⁎ *p* < 0.01.

Finally, the accuracy improvements produced by the methods introduced were put to the test by comparing them with results from the commonly used approach of using half the radius for travel distances, and constant intra-zonal speeds. Table 7 gives an overview of the results in the case of linear distance decay and category IV travel times. The accuracy results for category II and III travel times give way to the same conclusions (Appendix B). We find that using either the internal distances or speed models presented in this paper improves accuracy considerably, and errors are further reduced when those approaches are combined. Furthermore, the effects of scale in travel time estimation errors are much reduced by these methods. It is immediately clear that with the traditional method of using the ½ radius and a constant travel speed, estimation errors grow much more substantially in the case of larger areal units than is the case when using the methods proposed here.

**Table 7:** Accuracy of the results from the proposed method and Rich's (1978) method with constant travel speeds for all category IV travel times.

Distances	½ radius		Distance estimated by a nonlinear function of average distance to population-weighted centroids (Eq. (5.3))	
Speeds	Constant travel speed (24.3 km/h)	Non-linear travel speed (estimated by Eq. (7))	Constant travel speed (24.3 km/h)	Non-linear travel speed (estimated by Eq. (7))
*MAPE*				
LAU-2	0.191	0.195	0.109	0.093
LAU-1	0.440	0.229	0.228	0.113
GR	0.881	0.305	0.158	0.231
Total	0.227	0.200	0.123	0.097

*RMSE*				
LAU-2	1.964	2.007	1.283	1.086
LAU-1	7.210	3.896	3.821	2.178
GR	30.124	11.809	8.563	8.412
Total	4.447	2.617	1.981	1.546

Table note: Total values indicate average accuracy over all 342 observations.

## 5 Estimating extra-zonal interaction-weighted travel times

This section tackles source A errors, which are erroneous travel time estimations between two points that both represent spatially distributed interacting masses within a zone. We explore what is the best aggregated spatial representation of a spatially distributed mass, and whether the intra-zonal spatial distribution of the population will cause systematic deviations of interaction weighted travel times in any *i* to *j* relation. In addition, an alternative method of improving interaction weighted travel time accuracy is investigated, using multiple samples per o-d relation.

### 5.1 Existing methods

A literature review has determined that zones are usually represented by one node; usually either by the zone's geographic or population-weighted centroid. Most existing studies use the former node (Table 8), but some authors claim that this is not ideal. Miller (1996) doubts that the geographic centroid is accurate, in particular where there are complex boundaries or many geometrical differences between zones. Holl (2007) expects that large zones, in particular, cause inaccuracies. Hodgson et al. (1997) advocate gravity weighted centroids. None of the studies mentioned provides empirical proof.

**Table 8:** Review of existing approaches to estimate travel time between different areal units.

	Nodes	Penalties
Condeço-Melhorado et al. (2011)	Geographical centroids	½ internal travel time
Condeço-Melhorado et al. (2013)	Geographical centroids	½ internal travel time
Gutiérrez et al. (2011)	Geographical centroids	½ internal travel time
Chandra and Vadali (2014)	Geographical centroids	No
Holl (2007)	Geographical centroids	No
Jacobs-Crisioni and Koomen (2014)	Geographical centroids	No
Salas-Olmedo et al. (2014)	Geographical centroids	No
Kotavaara et al. (2011)	Population-weighted centroids	No

Regarding the question as to whether intra-zonal spatial distribution of activities causes systematic deviations, we find duality in the literature. Some studies assume that their single-point representation is sufficient, while others add a ‘penalty’ travel time to the centroid-to-centroid travel times (Table 8). In the latter case, the added travel time is supposed to reflect the intra-zonal distribution of activities or the time required to enter and leave a zone. No study has yet provided proof to support either choice.

### 5.2 Exploration of zone-specific effects

In order to improve estimates based on population-weighted centroids, a search of models was carried out in order to obtain variables that may be included as penalties. The dependent variable indicated differences between benchmark travel times and travel times between the population-weighted centroids,^3^ i.e. ∆* t*_*ij*_^*w*^ = *t*_*ij*_^*w*^ − *t*(*γ* = 1)_*ij*_^*IV*^. The variables used here are the same as the variables used in Section 4, supplemented by intra-zonal interaction-weighted travel times (*t*_*ii*_^*w*^ and *t*_*jj*_^*w*^). The following equation has been fitted on the LAU-2 travel time data in an OLS approach:

(10) $$\Delta t_{\mathrm{ij}}^{w}=\beta 0+\beta 1X_{i}+\beta 2X_{j}+\varepsilon_{\mathrm{ij}}.$$

Here, *X* indicates a specific variable that is available for the origin and destination zones. The results are given in Table 9 and indicate two important findings: all variables have highly significant effects, and all estimators are decidedly symmetrical, i.e. estimations are equally affected by the origin- and destination-characteristic. Nevertheless, despite the fact that many relevant variables have been established here, none of the selected variables is useful for improving the accuracy of interaction weighted travel-time estimates. The most accurate model improves accuracy from 2.625 to 2.525 min; i.e. by six seconds. We consider this hardly useful.

**Table 9:** Results of fitting Eq. (10). The results are sorted by ascending RMSE so that the most accurate results are at top.

Variable	*β*0	*β*1	*β*2	R^2^	RMSE
Area	0.573⁎⁎ (21.47)	− 0.005⁎⁎ (− 32.80)	− 0.005⁎⁎ (− 32.80)	0.02	2.525
Gini index	2.123⁎⁎ (30.10)	− 2.083⁎⁎ (− 27.54)	− 2.083⁎⁎ (− 27.54)	0.02	2.534
Population	− 0.543⁎⁎ (− 62.02)	− 0.000⁎⁎ (− 19.25)	− 0.000⁎⁎ (− 19.25)	0.01	2.545
Moran's I	− 0.082⁎⁎ (− 3.85)	− 0.851⁎⁎ (− 18.95)	− 0.851⁎⁎ (− 18.95)	0.01	2.545
WUP	-0.784⁎⁎ (− 71.43)	1.125⁎⁎ (18.76)	1.125⁎⁎ (18.76)	0.01	2.546
Network density	− 1.534⁎⁎ (− 42.06)	0.729⁎⁎ (18.65)	0.729⁎⁎ (18.65)	0.01	2.546
Intra-zonal travel times estimates	− 1.579⁎⁎ (− 23.47)	0.059⁎⁎ (10.39)	0.059⁎⁎ (10.39)	0.00	2.552
Ln (Population)	1.033⁎⁎ (7.87)	− 0.091⁎⁎ (− 8.83)	− 0.091⁎⁎ (− 8.83)	0.00	2.553
Degree of urbanisation	0.085 (1.38)	− 0.125⁎⁎ (− 7.96)	− 0.125⁎⁎ (− 7.96)	0.00	2.554
Population density	− 0.646⁎⁎ (− 66.71)	0.000⁎⁎ (7.08)	0.000⁎⁎ (7.08)	0.00	2.554

Table note: extra-zonal observations from LAU-2 matrix so that *N* = 89,700. ^⁎^*p* < 0.05

⁎⁎ *p* < 0.01.

**Fig. 5:**
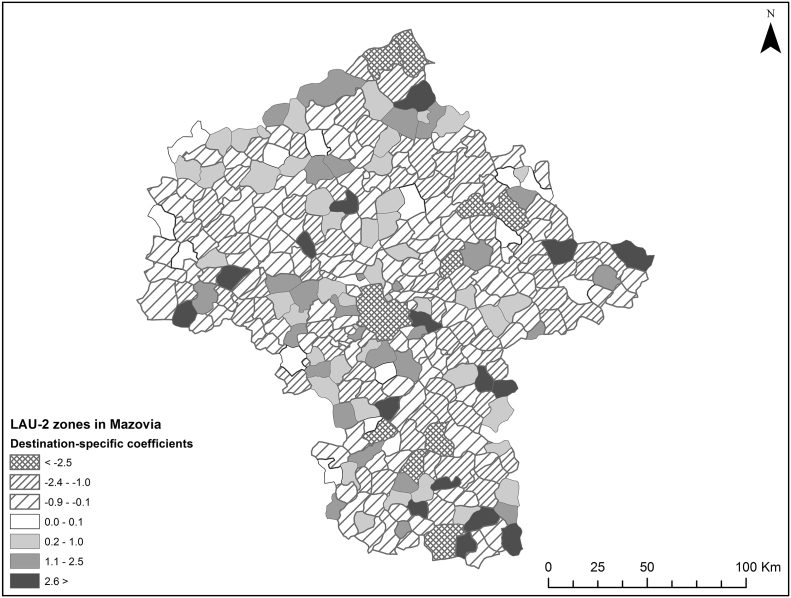
Estimated destination-specific coefficients.

More complex models have been attempted that included interactions with *d*_*ij*_^*w*^, but did not yield substantial improvements to model accuracy. Thus, the zone-specific variables tried do not provide much help in improving model accuracy. This begs the question whether differences in ∆* t*_*ij*_^*w*^ are even related to the terminating zones? To determine this, the values have been fitted on origin and destination-specific fixed effects, so that:

(11) $${∆t}_{\mathrm{ij}}^{w}={\beta 1_{i}X}_{i}+{\beta 2_{j}X}_{j}+\varepsilon_{\mathrm{ij}},$$

in which the data have been fitted into the above equation using an OLS approach. The variable *X* consists of a vector of dichotomous values that have the value 1 for one specific origin or destination, and 0 otherwise. *N* = 89,700. This exercise yielded an R^2^ of 0.69, an RMSE of 1.463 and, compared with the values in *t*_*ij*_^*IV*^, a MAPE of 0.014. Both origin and destination specific estimators were for the most part highly significant (generally *p* < 0.01). For all 300 LAU-2 zones, the origin-specific effects of a zone were almost perfectly symmetrical to the destination-specific effects of the same zone; the maximal difference between origin and destination specific effects was 0.1%.^4^ Thus, both origins and destinations have a completely similar, highly significant effect on ∆* t*_*ij*_^*w*^ and we indeed conclude that the values in ∆* t*_*ij*_^*w*^ depend largely on the terminating zones. Unfortunately, when mapped, the coefficients do not yield a recognisable pattern (see Fig. 5). Thus, our pessimistic conclusion is that zone-specific conditions do structurally affect travel time estimation errors, but a logical explanation for these errors remains elusive.

### 5.3 Proposed solutions

We identify two methods to reduce errors that come into play with interaction-weighted travel time and aggregated areal units. The first solution is using population-weighted centroids rather than geographically-weighted centroids. As Table 10 shows, travel times between population-weighted centroids provide a more accurate representation of interaction-weighted travel times between zones than travel times between geographic centroids. Surprisingly, the average differences are not monotonously positive or negative; thus, neither centroid methods seem to cause structural biases in the estimation procedure. This reinforces our conclusion that the existence of travel time errors must relate to the characteristics of each particular zone. We do find that population-weighted centroids have somewhat better average accuracy results in all cases except for the larger regions. The root mean square errors (RMSE) indicate that population weighted centroids, in particular, reduce the worst errors in a matrix, regardless of resolution. All in all, travel times between population-weighted centroids are most likely to produce more reliable approximations of interaction-weighted travel times.

**Table 10:** Accuracy of results from extra-zonal travel times using population-weighted and geographic centroids.

Centroid type	Geographically weighted			Population-weighted		
Indicator type	Average dif	MAPE	RMSE	Average dif	MAPE	RMSE
LAU-2	0.194	0.031	3.540	− 0.600	0.024	2.625
LAU-1	1.831	0.067	7.092	0.988	0.043	4.709
GR	− 2.785	0.074	10.064	− 6.099	0.079	9.376
Total	0.219	0.032	3.623	− 0.576	0.025	2.671

Table note: values in “average dif” indicate average differences between estimated and benchmark travel time values.

The second method to improve travel estimate accuracy is through the underlying interaction-weighted travel time distribution. We found that the 35th percentile travel time consistently yields a more accurate estimate than the population-weighted centroid approach (for evidence, see Appendix III). This begs the question whether sampling random travel times may improve estimated travel time accuracy? In order to find out if a sampling strategy can improve the population-weighted centroid approach, travel times have been selected from, depending on the case, between 2 and 50 random points in each areal unit. Subsequently from those samples, the values closest to the 35th percentile value have been selected and their accuracy compared with the benchmark values.

When comparing MAPE and RMSE results (Table 11) it becomes clear that, as can be expected, this approach becomes more reliable with larger samples. At the LAU-2 and greater regions levels, the 35th percentile value is slightly more accurate than using population-weighted centroids; while at the LAU-1 level, the accuracy of population-weighted centroids is only approximated with as many as 50 sample points per zone. Clearly, there is a trade-off here between accuracy gain and computational complexity. We leave the decision of whether the potentially increased accuracy is worth the added complexity to the analyst.

**Table 11:** Accuracy results of 35th percentile travel times of randomly selected sample points with different sample sizes, when compared with benchmark values.

Sample size	MAPE					RMSE				
	2	5	10	20	50	2	5	10	20	50
LAU-2	0.047	0.032	0.025	0.020	0.016	5.058	3.443	2.749	2.132	1.713
LAU-1	0.093	0.064	0.061	0.051	0.046	10.372	6.669	6.671	5.457	4.901
GR	0.156	0.094	0.081	0.064	0.066	19.072	11.698	10.205	8.481	8.348
Total	0.048	0.032	0.026	0.020	0.017	5.186	3.518	2.853	2.223	1.808

Table note: the selected travel times are the values closest to the 35th percentile value of sample-to-sample interactions. Thus, with a sample of 2 per LAU2, this value is generally 0.35(2^2^) ≅ 1; with a sample of 5 per LAU2, this value is generally 0.35(5^2^) ≅ 9; etc.

## 6 Closing remarks

This article explores the impacts of spatial aggregation on SIMs and potential accessibility that exist even in fully controlled environments. We find that, under aggregation, arithmetic averages are not sufficient to obtain reliable benchmark travel times. Instead, it is necessary to use harmonic means. This enables us to determine straightforward travel times between pairs of points and interaction-weighted travel time. The latter additionally entails a measure of propensity to produce interactions at the origins and destination. Furthermore, the degree of separation between them is influenced by any applied impedance function. We find that interaction-weighted travel times vary from simple centroid-to-centroid travel times, both if only one termination point represents the aggregated interacting masses, or if both termination zones are aggregated. In this paper, harmonic mean travel times are used as benchmark values to evaluate different methods of estimation of interaction-weighted travel times for intra-zonal and extra-zonal trips.

We select a set of easily computable variables to explore whether they can be used to estimate accurate interaction-weighted travel times without the tedious task of measuring travel times between every single pair of origin-destination nodes at a very fine resolution. The best fitting variable – the population-weighted average distance to a zone's population-weighted centroid – is substantially more accurate than any other variable. In fact, the proposed method halves the mean estimation error, compared to the most commonly used method (i.e. Frost and Spence's ½ radius). Moreover, the proposed method reduces the scale effects of the MAUP, as estimation errors from the proposed method increase less substantially than would be the case if area-based methods are used to obtain intra-zonal distances. Lastly, this variable has a limited computational complexity and can be computed with any GIS software. The necessary fine resolution population data are freely available. Thus, our first main result is that in the case of spatial interaction data, weighted average distances to the gravity centroid should be used to estimate intra-zonal interaction-weighted distances.

Using the proposed method and a fixed travel speed gives more accurate intra-zonal travel times. In addition, we propose a model to take into account the distinctly non-linear increase of effective travel speeds with larger Euclidean distances between origin and destination nodes. The case-specific model applied is assumed to be uniform for the whole study area. It yields reasonably accurate results, and in any case can be obtained from the travel time matrix that is necessary for accessibility or SIM applications. We must nevertheless acknowledge that the introduced speed model can be further improved, for example by using observed vehicle trajectories that might provide more accurate and tailored estimators, or with more refined approaches that, for example, take into account travel costs or variation in travel speeds by geography (e.g. by origin and destination region) or by time-of-day (e.g. peak and off-peak).

We find a lack of consensus between researchers on the merits of using either geographic or population-weighted centroids as the nodes that represent areal units. Existing studies use one or the other, but, to the best of our knowledge, no-one provides a quantitative argument for that decision. Our results show that the population-weighted centroids provide more accurate interaction-weighted travel times and should be preferred over geographic centroids. Alternatively, the 35th percentile value of a sample of travel times can be used to obtain more accurate travel times. Finally, we explore estimations of extra-zonal interaction-weighted travel times. Even though zone-specific conditions do strongly affect extra-zonal interaction weighted travel times, we find no variables that can function as useful penalties at the origin or destination. Therefore, we recommend not using any such penalties in SIM or potential accessibility applications. More research will be needed to better identify proxies for the zone-specific effects found.

All in all, the methods outlined in this paper considerably improve the interaction-weighted travel time estimation methods that are needed in cases of spatial interaction with spatially aggregated data. The paper has focused on scale effects with entirely modelled data. The shape effects of zonal units may also have an additional effect on the findings, but these mostly come into play when the study population displays heterogeneous behaviour and the zone boundaries coincide with relatively homogeneous groups (Jacobs-Crisioni et al., 2014). Thus, shape issues may be more important in the case of observed spatial interaction than in the modelled data used in this study - in which variance only depends on the variables included. Other implications of our findings surely need further analysis. We find an inherent relationship between impedance functions and interaction-weighted travel times. That relationship creates an endogeneity problem when empirically fitting a spatial interaction model, potentially requiring convergence methods to obtain a fit of the final impedance function. Lastly, these findings have been obtained assuming unconstrained SIMs and potential accessibility measures. More work will be needed to understand how interaction-weighted aggregate travel times are affected by origin and/or destination constraints, as described by, for example, Alonso (1978) and Geurs and Ritsema van Eck (2003).

## Figures and Tables

**Table B1:** Results of fitting intra-zonal distance models as in Eq. (8) with *d*_*i* = *j*_^*II*^ and *d*_*i* = *j*_^*III*^.

Impedance function (value)	*β*0 (z-score)	*β*1 (z-score)	R^a^	RMSE (km)
Power function (− 1.00)	0.72971⁎⁎ (37.85)	0.98037⁎⁎ (97.61)	0.9672	0.7544
Power function (− 1.25)	0.71009⁎⁎ (35.47)	0.96761⁎⁎ (89.44)	0.9624	0.7643
Power function (− 1.50)	0.69939⁎⁎ (34.09)	0.94818⁎⁎ (83.04)	0.9587	0.7542
Power function (− 1.75)	0.69898⁎⁎ (33.51)	0.92058⁎⁎ (77.62)	0.9564	0.7269
Power function (− 2.00)	0.70947⁎⁎ (33.56)	0.88416⁎⁎ (72.64)	0.9555	0.6861
Power function (− 2.50)	0.75739⁎⁎ (34.98)	0.79053⁎⁎ (62.96)	0.9574	0.5848
Power function (− 4.00)	0.90694⁎⁎ (41.54)	0.53233⁎⁎ (41.65)	0.9706	0.3541
Power function (− 8.00)	0.98728⁎⁎ (67.10)	0.26989⁎⁎ (29.25)	0.9899	0.1466
Exponential (− 0.006)^b^	1.06078⁎⁎ (235.42)	0.97601⁎⁎ (602.53)	0.9991	0.1748
Exponential (− 0.023)^b^	1.09129⁎⁎ (198.09)	0.94840⁎⁎ (482.73)	0.9987	0.2026
Exponential (− 0.039)^c^	1.12057⁎⁎ (158.90)	0.92309⁎⁎ (369.80)	0.9980	0.2469
Exponential (− 0.068)^d^	1.17131⁎⁎ (116.61)	0.87991⁎⁎ (250.33)	0.9961	0.3233
Exponential (− 0.15)	1.27533⁎⁎ (79.90)	0.78586⁎⁎ (142.44)	0.9915	0.4272
Exponential (− 0.25)	1.36790⁎⁎ (65.47)	0.68297⁎⁎ (93.77)	0.9874	0.4562
Exponential (− 0.289)^a^	1.34899⁎⁎ (68.17)	0.70712⁎⁎ (102.94)	0.9883	0.4533
Exponential (− 0.90)	1.38183⁎⁎ (51.42)	0.49976⁎⁎ (47.37)	0.9809	0.4090

Table note: the intra-zonal observations come from all areal units so that *N* = 342. ^⁎^p < 0.05. Exponential function selected by the authors, except values obtained from:

a Haynes et al. (2003).

b Rosik et al. (2015).

c Geurs and Ritsema van Eck (2001).

d Martínez and Viegas (2013).

⁎⁎ *p* < 0.01.

**Table B2:** Accuracy of the results from the proposed method and Rich's (1978) method with constant travel speeds for all category II and III travel times.

Distances	½ radius		Distance estimated by a nonlinear function of average distance to population centroids (Eq. ((5.1), (5.2))	
Speeds	Constant travel speed (24.3 km/h)	Non-linear travel speed (estimated by Eq. (7))	Constant travel speed (24.3 km/h)	Non-linear travel speed (estimated by Eq. (7))
*MAPE*				
LAU-2	0.241	0.296	0.176	0.149
LAU-1	0.659	0.439	0.311	0.181
GR	1.228	0.569	0.548	0.078
Total	0.299	0.315	0.196	0.152

*RMSE*				
LAU-2	2.177	2.526	1.872	1.601
LAU-1	8.769	5.871	4.411	2.704
GR	33.708	16.456	20.185	3.418
Total	5.098	3.549	3.163	1.788

**Fig. C1:**
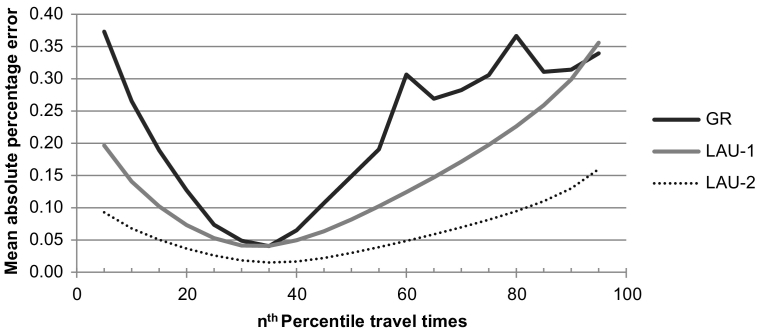
Mean absolute percentage errors for 5th to 95th percentile travel times meant to represent interaction-weighted travel times between two zones.

**Fig. C2:**
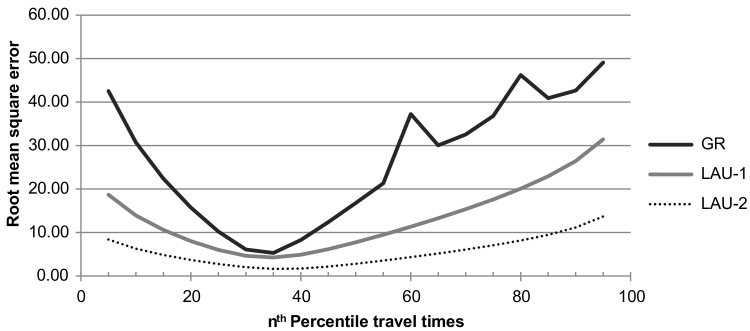
Root mean square errors for 5th to 95th percentile travel times meant to represent interaction-weighted travel times between two zones.

## Appendix A Derivation of harmonic mean travel times in the case of distance decay

As noted in Section 2, the interaction-weighted travel times for potential interaction at *a* depend on the distance decay function imposed on the interaction between those people. Let's reuse the simplest explanation offered in Section 2, with four people living in different destinations that are respectively 4, 6, 7 and 7 min away from our point of interest *a*. As the distance decay function, let's choose $f\left(t_{\mathrm{ab}}\right)={t_{\mathrm{ab}}}^{2}$. To obtain accurate accessibility values after aggregation, the harmonic mean result of distance decayed travel times can then be obtained through:

(A1) $${\left(t_{\mathrm{ab}}^{\mathrm{II}}\right)}^{2}=\frac{\sum P_{b}}{\sum \frac{P_{b}\;}{{t_{\mathrm{ab}}}^{2}}}=\frac{4}{\left(\frac{1}{7^{2}}+\frac{1}{7^{2}}+\frac{1}{6^{2}}+\frac{1}{4^{2}}\right)}≅30.512$$

Potential interactions at *a* can then be calculated as *A*_*a*_ = ∑ *P*_*b*_/(*t*_*ab*_^*II*^)^2^ ≅ 4/30.512. To obtain benchmark travel times, the harmonic mean distance decay function results have been inverted into interaction weighted travel times by using the reciprocal of the distance decay function. In this case *t*(*γ* = 2)_*ab*_^*II*^ ≅ 30.512^1/2^ ≅ 5.524 minutes. This value is slightly lower than the 5.695 min computed for *γ* = 1 (see Section 2). The logic here is that, with ever sharper distance decay functions, the closest person in the aggregated unit contributes an ever larger share to total interaction opportunity. Lastly, this example uses a power distance decay function, for which the inverse is computed as *x*^1/*γ*^. For exponential functions, the inverse is computed as ln*x*/*δ*.

## Appendix C Travel time accuracy with percentile travel times from a more detailed matrix

In this appendix, the accuracy of results is given for cases in which a percentile travel time from the travel times between disaggregated origins and destinations is used to represent the travel times between the zones in which those disaggregate origins and destinations are located. In all cases, the percentile travel times are used to approximate *t*(*γ* = 1)_*ij*_^*IV*^. Fig. C1 shows the MAPE results for several percentile values; while Fig. C2 shows the RMSE results.

## Footnotes

1 Hillsman and Rhoda (1978) also identified Source C errors – when the use of an aggregated spatial unit causes individuals to be related to the ‘wrong’ facility in discrete allocation methods. Hodgson et al. (1997) also propose Source D errors, related to the application of discrete selection algorithms for continuous space. We consider both types of error irrelevant for continuous spatial-interaction modelling exercises.

2 First, the population-weighted centroids of all zones are obtained. In geographically projected data, this is easily done by obtaining the weighted mean *x* and *y* values of all populated points, so that ${\mathrm{wx}}_{i}=\sum_{a\in I}^{n}P_{a}x_{a}/\sum_{a\in I}^{n}P_{a}$ and ${\mathrm{wy}}_{i}=\sum_{a\in I}^{n}P_{a}y/\sum_{a\in I}^{n}P_{a}$, yielding the points *w*_*i*_ (*wx*_*i*_, *wy*_*i*_). Then the distance for each point in *a* to *w*_*i*_ is easily obtainable using the Pythagorean theorem, and is expressed here as *dw*_*a*_; subsequently the weighted average population distance is computed as $\overset{-}{{\mathrm{dw}}_{i}}=\sum_{a\in I}^{n}P_{a}dw_{a}/\sum_{a\in I}^{n}P_{a}$. This variable was computed using the open-source GeoDMS software (ObjectVision, 2014), but can be easily computed in other GIS packages.

3 Population-weighted centroids were used as a basis here, because those yield less errors than geographic centroids. Proof of the higher accuracy of population-weighted centroids is given in a later section of this paper.

4 To preserve space, the 600 estimated coefficients are not listed here. They are visible in Fig. 5 and available on request.
